# Antimicrobial Resistance Patterns and Biofilm Analysis via Sonication in Intensive Care Unit Patients at a County Emergency Hospital in Romania

**DOI:** 10.3390/ph18020161

**Published:** 2025-01-25

**Authors:** Ioana Roxana Codru, Bogdan Ioan Vintilă, Alina Simona Bereanu, Mihai Sava, Livia Mirela Popa, Victoria Birlutiu

**Affiliations:** 1 Faculty of Medicine, Lucian Blaga University, 2A, Lucian Blaga Str., 550169 Sibiu, Romania; ioanaroxana.bera@ulbsibiu.ro (I.R.C.); alina.bereanu@ulbsibiu.ro (A.S.B.); mihai.sava@ulbsibiu.ro (M.S.); liviamirela.popa@ulbsibiu.ro (L.M.P.); victoria.birlutiu@ulbsibiu.ro (V.B.); 2 County Clinical Emergency Hospital, 2–4, Corneliu Coposu Bld., 550245 Sibiu, Romania

**Keywords:** ventilator-associated pneumonia, antimicrobial resistance, biofilm, endotracheal tube, ESKAPEE, ESKAPE pathogens, MDR bacteria, sonication, ICU

## Abstract

**Background/Objectives:** Ventilator-associated pneumonia (VAP) remains a critical challenge in ICU settings, often driven by the biofilm-mediated bacterial colonization of endotracheal tubes (ETTs). This study investigates antimicrobial resistance patterns and biofilm dynamics in ICU patients, focusing on microbial colonization and resistance trends in tracheal aspirates and endotracheal tube biofilms at a county emergency hospital in Romania. **Methods:** We conducted a longitudinal analysis of ICU patients requiring mechanical ventilation for more than 48 h. Tracheal aspirates and ETT biofilms were collected at three key time points: T1 (baseline), T2 (48 h post-intubation with ETT replacement), and T3 (92–100 h post-T2); these were analyzed using sonication and microbiological techniques to assess microbial colonization and antimicrobial resistance patterns. **Results:** In a total of 30 patients, bacteria from the ESKAPEE group (e.g., *Klebsiella pneumoniae*, *Acinetobacter baumannii*, *Staphylococcus aureus*) dominated the microbiota, increasing their prevalence over time. Resistance to carbapenems, colistin, and vancomycin was notably observed, particularly among *K. pneumoniae* and *A. baumannii*. Biofilm analysis revealed high persistence rates and the emergence of multidrug-resistant strains, underscoring the role of ETTs as reservoirs for resistant pathogens. The replacement of ETTs at T2 correlated with a shift in microbial composition and reduced biofilm-associated contamination. **Conclusions:** This study highlights the temporal evolution of antimicrobial resistance and biofilm-associated colonization in a limited number of ICU patients (30 patients). The findings support implementing routine ETT management strategies, including scheduled replacements and advanced biofilm-disruption techniques, to mitigate VAP risk and improve patient outcomes.

## 1. Introduction

In critically ill patients, mechanical ventilation (MV) is a crucial intervention widely utilized to support respiratory function. However, extended MV is associated with various complications, with ventilator-associated pneumonia (VAP) standing out as a significant cause of morbidity and mortality [1,2]. VAP develops due to a bacterial colonization of the lower respiratory tract, often aided by biofilm formation on the surface of the endotracheal tube (ETT) [3,4]. This colonization contributes to the pathogenesis of VAP and complicates the clinical management of mechanically ventilated patients [5].

It is no news that patients admitted to the intensive care unit (ICU) are 5–10 times more prone to infections than patients outside the hospital. Also, nosocomial infections increase morbidity and mortality rates, prolong hospitalization, and increase treatment costs [6,7,8]. Implementing infection control procedures in the ICU is one of the most crucial steps in preventing colonization and infection by multidrug-resistant (MDR) bacteria. The prolonged and frequent use of antibiotics in ICUs is one of the most significant factors contributing to the higher prevalence of MDR microorganisms in this setting [6,7,9].

Biofilm formation is incriminated in the appearance and persistence of ventilator-associated pneumonia. Sonication is an effective technique for dislodging bacteria from biofilms, making it valuable for analyzing pathogens on medical devices like endotracheal tubes. In ICU settings, biofilms on ETTs can conceal resistant organisms that contribute to persistent infections, complicating the treatment of ventilator-associated pneumonia. By disrupting these biofilms, sonication allows for a more accurate detection of the pathogens within, providing critical insights into the microbial load and resistance profiles. This process is particularly useful for understanding the role of biofilms in antimicrobial resistance in high-risk environments like the ICU [10,11,12,13].

The process of bacterial colonization in mechanically ventilated patients is intricate and affected by various factors. These factors include the length of intubation, the patient’s underlying medical condition, and the use of antibiotics [14,15,16]. Research has demonstrated that biofilm can form on endotracheal tubes within hours of intubation, creating a place where harmful bacteria can grow and spread to the lungs. The types of bacteria found on the ETTs and in tracheal aspirates change over time, starting with harmless organisms and later being replaced by drug-resistant pathogens [3,17]. Alteration in microbial composition over time can contribute to the onset of VAP, particularly in patients undergoing prolonged ventilation [18,19,20,21].

Numerous international organizations offer guidance on diagnosing and preventing ventilator-associated pneumonia. Most preventive measures focus on mechanical interventions, hand hygiene, and, arguably the most crucial, antibiotic stewardship methods. Despite the significant scientific interest in this area, gaps still need to be addressed to enhance our understanding and improve practices when caring for critically ill patients [22,23].

The primary objective of this study is to examine the temporal dynamics of microbial colonization and antibiotic resistance in mechanically ventilated ICU patients. We focus on microorganisms isolated from tracheal aspirates and biofilms on endotracheal tubes over sequential time points. By investigating microbial presence at key stages of MV, we aim to identify patterns of persistence, clearance, and the emergence of new organisms.

This study aims to address a significant gap in managing infections in the ICU. Specifically, interventions such as replacing endotracheal tubes to reduce the risk of ventilator-associated pneumonia and their effects on microbial ecology and resistance patterns in the ICU have not been thoroughly explored. Gaining insight into these dynamics could be valuable, as it may help to determine whether these interventions affect microbial colonization and resistance.

This study investigates the interplay between biofilm dynamics and trends in antimicrobial resistance, underscoring their intrinsic connection. Biofilm formation on ETTs not only facilitates microbial colonization but also creates a protective microenvironment that fosters the persistence of resistant pathogens. The dual focus of this research is to explore the challenges associated with ventilator-associated pneumonia (VAP) by examining the potential role of biofilm development in antimicrobial resistance. While this integrated approach highlights the importance of multifaceted strategies, such as advanced biofilm-disruption techniques and rigorous antimicrobial stewardship, it is crucial to consider the limited sample size of 30 patients and the monocentric nature of the study. These factors may influence the generalizability of our findings, and further research is needed to validate these insights in larger, more diverse populations.

The findings from this study aim to enhance our understanding of microbial behavior in mechanically ventilated patients, guiding future practices in ICU settings to improve patient outcomes and reduce the incidence of VAP.

## 2. Results

A total of 58 patients were initially considered for inclusion in this study. However, after the screening process, nearly half of them—28 patients—were excluded because they did not meet the eligibility criteria. Specifically, five patients had not signed the informed consent, eight patients had been ventilated for less than 48 h, and fifteen were deemed clinically unsuitable for the tube exchange at the scheduled time due to poor oxygenation or significant hemodynamic instability. Among the patients in the ICU who met the study criteria, the ages ranged from 33 to 83 years, with a mean age of 64, and the majority of patients were male (70%).

**Figure 1:**
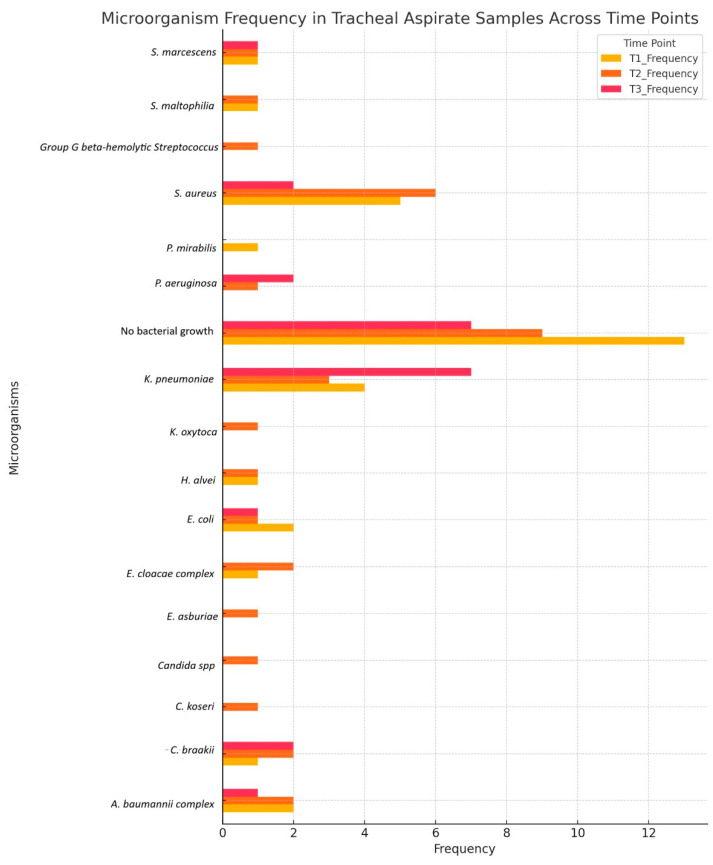
Prevalence of microorganisms in tracheal aspirates over time (T1, T2, T3).

**Figure 2:**
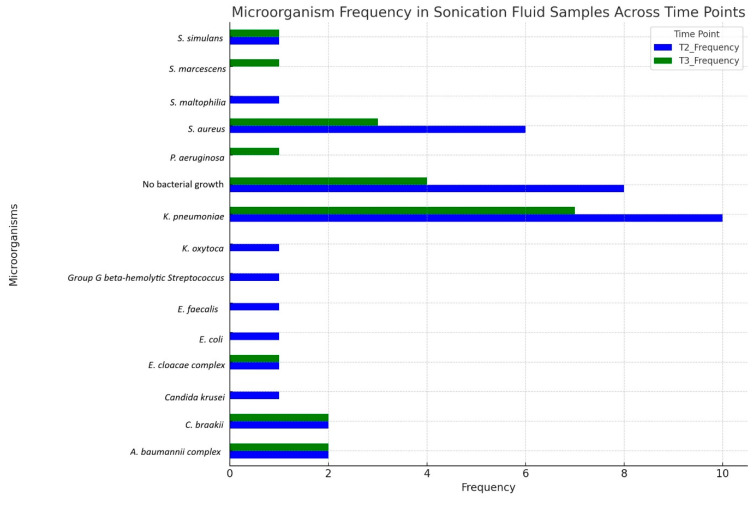
Prevalence of microorganisms in sonication fluid over time (T2, T3).

In our hospital’s microbiology laboratory, were cultivated various pathogens from patients’ tracheal aspirates and biofilms that developed on the tracheal tubes from patients admitted to ICU. (see Figure 1 and Figure 2). To better understand the prevalence of microorganisms at different time points, we have combined tracheal aspirates from T1, T2, and T3 into one figure and T2 and T3 sonication fluid into another.

In the study involving 30 patients, 13 displayed sterile tracheal aspirates at intubation, representing 43.33% of the cases and making it the most common finding. However, over time, the percentage of patients showing no bacterial growth in their tracheobronchial aspirates decreased to 30% at T2 and 35% at T3. In contrast, bacteria from the ESKAPEE (*Enterococcus faecium*, *Staphylococcus aureus*, *Klebsiella pneumoniae*, *Acinetobacter baumannii*, *Pseudomonas aeruginosa*, *Enterobacter* spp., and *E. coli*) group were frequently isolated, appearing in many tracheal aspirates. At T1, out of the 20 pathogens isolated, 13 (65%) belonged to the ESKAPEE group. This incidence increased with time, reaching 80% by T3. In addition to the main group of bacteria, other pathogens were isolated from the patients’ tracheal aspirates, although their frequency was significantly lower. These included *S. marcescens, S. maltophilia, C. braakii, E. faecalis*, other *Klebsiella* species, and various fungal species.

In the sonication liquid obtained from the intubation specimen after exchange and cultivation on culture media, pathogens of the same species were identified; however, their incidence differed from that found in the tracheal aspirates. At T2, bacteria containing biofilms were diagnosed in 90% of cases after the biofilms were dislodged from the ETTs and the sonication fluid cultivated. Among the pathogens identified, bacteria from the ESKAPEE group represented 74%. In contrast, at T3, biofilm was observed in 77.77% of the cannula specimens, but the incidence of the ESKAPEE group decreased to 55.55%.

**Figure 3:**
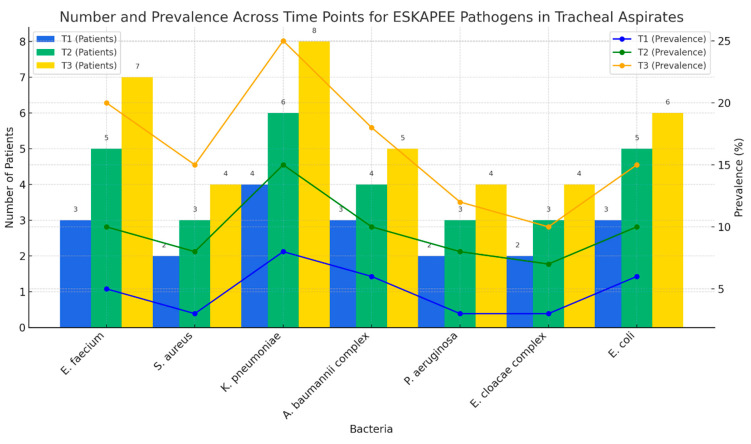
ESKAPEE bacteria—number and prevalence (tracheal aspirates).

It is important to note that *K. pneumoniae* shows the most significant upward trend at the specified time points and has the highest prevalence in the tracheal secretions of patients who were mechanically ventilated for more than six days. This bacterium accounts for half of the bacteria in the ESKAPEE group, representing 54% and 35% of the total number of pathogens identified. When describing the second most-frequent pathogen, *S. aureus*, identified in our patients, we can observe a peak at T2 before it declined. Out of five strains of *S. aureus*, at T1, two of them were MRSA. At T2, four strains had a high antibiotic resistance, being methicillin resistant. The patients that still presented clinical signs of VAP at T3 also had positive tracheal aspirates for MRSA (Figure 3).

**Figure 4:**
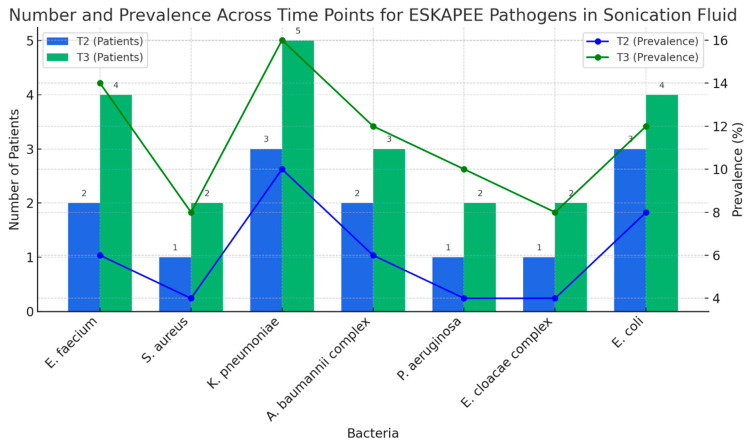
ESKAPE bacteria—number and prevalence (sonication fluid).

Our analysis of the sonication fluid from patients revealed a decrease in the detection frequency of K. pneumoniae in biofilms from time point T2 to T3. Despite this decrease, *K. pneumoniae* remains a significant concern (Figure 4).

**Figure 5:**
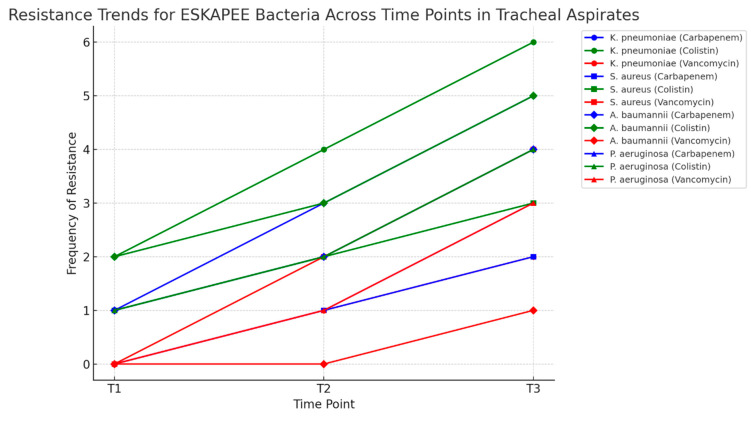
Resistance trends—tracheal aspirates.

We analyzed the antimicrobial resistance of all the pathogens identified in the tracheal aspirates, with the most significant findings occurring within the ESKAPEE bacteria group. This analysis was conducted at the set time points and focused on three critical antibiotics used for severely ill patients: carbapenems, vancomycin, and colistin. (Figure 5). The frequency of appearance is represented on the *Y*-axis, while the different time points are shown on the *X*-axis. Each line represents the frequency of resistance observed for key pathogens to critical antibiotics. A trend analysis of antimicrobial resistance revealed distinct patterns among ESKAPEE pathogens in tracheal aspirate samples over time. *K. pneumoniae* showed the most pronounced increase, with carbapenem and colistin resistance rising significantly from T1 to T3. The same applies to the *A. baumannii complex* and *P. aeruginosa*, which gained carbapenem resistance by T3. *S. aureus* also showed increased resistance, with vancomycin resistance developing by T3.

**Figure 6:**
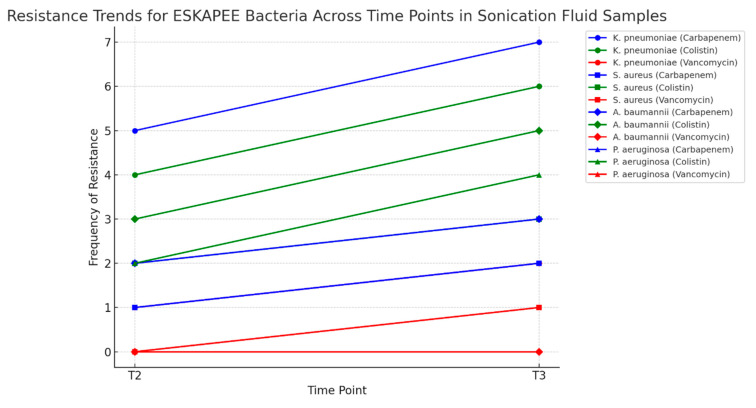
Resistance trends—sonication fluid.

In the sonication fluid, antibiotic resistance was observed, particularly in *K. pneumoniae* and the *A. baumannii* complex, both of which developed resistance to carbapenems and colistin between the two time points. Additionally, resistance to vancomycin was noted in *S. aureus*, showing a slight upward trend (Figure 6).

While the primary focus of the study was on the three antibiotics (Colistin, Vancomycin, and carbapenems), it is noteworthy that the combination of Ceftazidime and Avibactam exhibited ineffectiveness against certain strains of *K. pneumoniae*. In patients demonstrating this form of extended antibiotic resistance, the underlying mechanisms include the presence of New Delhi metallo-beta-lactamase (NDM), Klebsiella pneumoniae carbapenemase (KPC), and OXA-48-like enzymes. Among the 13 *K. pneumoniae* strains cultured from tracheal aspirates, 6 exhibited resistance to the majority of antibiotics tested, including those considered last-resort options, such as Ceftazidime/Avibactam. This particularly resistant strain was isolated from two patients, leading to the inability to successfully treat ventilator-associated pneumonia (VAP) in these individuals, irrespective of the antibiotic combinations administered. Upon analyzing the sonication fluids, we noted a significantly higher incidence of *K. pneumoniae* colonization within biofilms compared to tracheal aspirates. During the two designated time points in which sonication was performed on the endotracheal tube, *K. pneumoniae* was detected in 17 instances, of which 13 exhibited resistance to Ceftazidime/Avibactam. These observations suggest that implementing scheduled tube changes may effectively limit the dislodgement of bacteria from biofilms, thereby mitigating their migration into the lungs and reducing ventilator-associated pneumonia.

The other predominant bacterium isolated from patients in our intensive care unit (ICU) was Staphylococcus aureus, identified in 14 tracheal aspirates and 8 biofilm samples from endotracheal tubes (ETTs). The sensitivity profile of this bacterium remained consistent across the various time points at which samples were collected. In all instances, S. aureus exhibited susceptibility to Linezolid, Teicoplanin, Ceftaroline, Cotrimoxazole, Rifampicin, and Doxycycline.

Following a descriptive analysis of the pathogens’ antibiotic resistance, we conducted a statistical evaluation to identify any significant changes in antibiotic resistance with the same antibiotics at various time points for both tracheal aspirates and sonication fluid. Notably, the endotracheal tube was replaced before the collection of each specimen. The null hypothesis states that there is no significant change in antibiotic resistance before and after tube exchange, while the alternative hypothesis suggests a statistical difference exists. As the data are not normally distributed, a Wilcoxon Signed-Rank Test was conducted. Comparisons were made between different time points for the tracheal aspirate (T1 vs. T2, T2 vs. T3, T1 vs. T3) and the sonication fluid (T2 vs. T3). The results of this test did not reach statistical significance for any of the comparisons made (*p* > 0.05), although there are observable changes in resistance trends over time. However, colistin resistance shows a possible trend towards significance from T1 to T2 (*p* = 0.08), suggesting some degree of increase.

A statistical analysis was performed on the most common pathogens identified: Klebsiella pneumoniae, Acinetobacter baumannii, and Staphylococcus aureus. We focused on their resistance to carbapenems, colistin, and vancomycin over specific time points. We employed the Wilcoxon Signed-Rank Test for each pathogen to evaluate statistically significant variations in microbial resistance. The most pronounced finding was a notable increase in resistance to carbapenems for both *K. pneumoniae* and *A. baumannii*, with a *p*-value of less than 0.05 observed when comparing tracheal aspirates (T1 vs. T2, T1 vs. T3). Furthermore, a significant rise in resistance levels was recorded in the sonication fluid for *A. baumannii*. In terms of resistance to colistin, an ascending trend was identified; however, this increase did not reach statistical significance. Additionally, our analysis of *S. aureus* revealed the emergence of vancomycin resistance in tracheal aspirates, which was statistically significant with a *p*-value of less than 0.05, although no similar change was observed in the sonication fluid.

**Figure 7:**
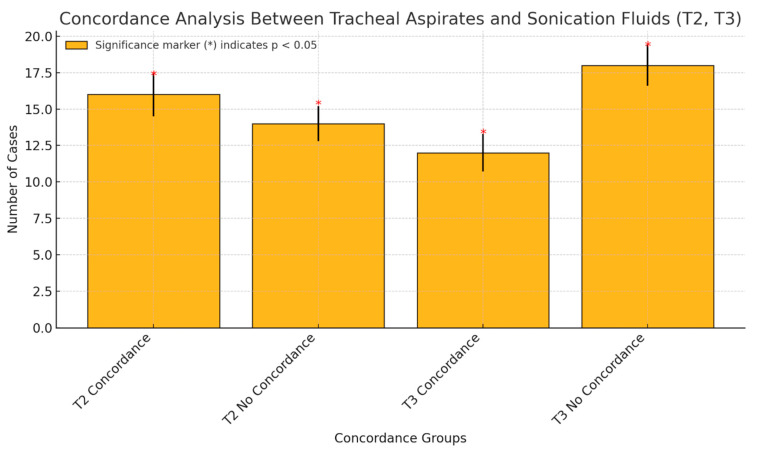
Concordance analysis (tracheal aspirates—sonication fluids, T2, T3).

Comparisons were made between different time points to identify potential variations in pathogen species cultured from tracheal aspirates and sonication fluid. In the comparison between T1 and T2, 20 cases showed the presence of the same microorganism, while 10 cases exhibited a change. In contrast, during the T2-T3 comparison, 12 patients maintained the same microorganism, while 18 patients showed a variation. Notably, the initial change in the endotracheal tube corresponded to a more significant variation between T2 and T3 than between T1 and T2 (Figure 7).

In conducting a concordance analysis of tracheal aspirates and sonication fluid at the T2 and T3 time points, following the replacement of the endotracheal tube, the findings revealed variations in the specimens collected and the sonicated ETT. At the T2 time point, 16 cases demonstrated concordance between the tracheal aspirates and the sonication fluid, whereas 14 cases exhibited discordance. Conversely, at the T3 time, there were 12 concordances and 18 instances of discordance. This observed decline in concordance suggests that changing the ETT may mitigate the risk of cross-contamination between tracheal secretions and the ETT.

We examined the dynamics of microorganisms over time and assessed whether these changes were statistically significant. A chi-square test revealed a statistically significant difference (*p*-value = 0.016). This result suggests that the types of microorganisms present at Time 1 (T1) are not evenly distributed across T2 and T3.

Our next objective is to see if the microorganisms present in the sonication fluid at T2 match or significantly differ from those found in the tracheal aspirates at T3. If there is a significant reduction or difference, it would indicate that changing the tube at T2 might effectively prevent biofilm contamination.

We used chi-squared tests to compare these groups and determine if the differences were statistically significant. When comparing T2 vs. T3 tracheal aspirate, we found a statistically significant difference (*p*-value < 0.05) between the microorganisms. This suggests that the microbial profile changes significantly after the tube is changed.

The biofilm on the EET specimen at T2 (T2 sonication fluid) vs. T3 tracheal aspirate showed another statistically significant difference between the microorganisms found in the sonication fluid of the tube at T2 and the tracheal aspirates at T3 (*p*-value: 0.00015). This supports the idea that changing the tube at T2 might effectively prevent the biofilm from contaminating tracheal secretions later.

**Figure 8:**
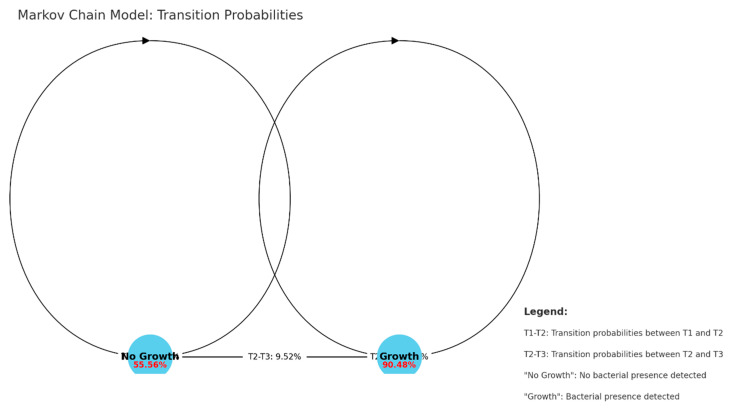
Markov Chain Model: transition probabilities.

In our final analysis of the data collected from patients admitted to the ICU, we performed a Markov Chain Analysis to assess the persistence and clearance of microorganisms (see Figure 8). This model allowed us to quantify the likelihood of bacterial presence or clearance over time in patients undergoing MV. We categorized the data from tracheal aspirates and sonication fluid samples collected at three-time points (T1, T2, and T3) into two states: No Growth (0)—indicating no bacterial growth detected, and Growth (1)—indicating any bacterial growth observed. We calculated the transition probabilities from T1 to T2 and from T2 to T3. The results from the T1 to T2 transition matrix were as follows: No Growth/No Growth (no microorganisms detected in both T1 and T2) occurred in 71.43% of cases; No Growth/Growth was found in 28.57%; Growth/No Growth was observed in 16.67%; and Growth/Growth occurred in 83.33%. For the T2 to T3 transition matrix, the results were as follows: No Growth/No Growth was seen in 85.71%; No Growth/Growth was detected in 14.29%; Growth/No Growth occurred in 20%; and Growth/Growth was present in 80%.

This analysis offers valuable insights into microbial dynamics in mechanically ventilated patients, highlighting several critical trends: a high persistence rate, the emergence of microorganisms over time, and a low probability of intervention effectiveness.

## 3. Discussion

ESKAPEE is an extended group of aggressive pathogens, including *E. coli*, which is highly prevalent in ICU settings. Hospital-related infections caused by these pathogens often pose a high risk of morbidity and prolongation of hospital stay, especially in mechanically ventilated patients [21,24,25,26].

This study focused on bacterial pathogens from the ESKAPEE group. While these organisms account for a significant proportion of ICU-acquired infections, incorporating viruses and fungi in future research would provide a more comprehensive understanding of microbial dynamics in critically ill patients.

In our study, *K. pneumoniae*, *A. baumannii complex*, and *S. aureus* were identified as the most prevalent ESKAPEE pathogens in the intensive care unit setting, with their frequency exhibiting a notable increase across the three sampled time points (T1, T2, and T3). *K. pneumoniae* demonstrated the highest detection frequency, particularly in later tracheal aspirate samples, suggesting its potential for rapid colonization or infection in mechanically ventilated patients. The presence of these pathogens in tracheal aspirates suggests their role in the pathogenesis of ventilator-associated pneumonia, particularly in its late-onset form. The observed high prevalence of these pathogens is consistent with their recognition as leading causes of healthcare-associated infections in ICU environments. Such settings often impose selective pressures that favor the proliferation of highly resilient and opportunistic pathogens, particularly in patients with compromised immune systems and those experiencing extended hospitalizations [24,27,28,29,30]. Our results align with the existing literature, highlighting the necessity for routine pathogen surveillance in ICU settings to facilitate early detection and intervention.

The higher detection rates in tracheal aspirates could suggest that these pathogens are adept at colonizing the respiratory tract, potentially contributing to ventilator-associated infections. Regular tracheal sampling, alongside the careful monitoring of biofilm formation on ETTs, could enhance the detection and management of these infections.

The data indicated a progressive increase in ESKAPEE pathogens, especially *K. pneumoniae*, from T1 to T3 in tracheal aspirates. This trend may reflect the risk of pathogen accumulation with prolonged ICU stays and factors such as antibiotic exposure, invasive devices, and cross-contamination. This temporal trend highlights the importance of early and consistent monitoring for ESKAPEE pathogens in patients with extended ICU admissions. Implementing time point-based sampling may help in detecting pathogens earlier, thereby enabling timely intervention and potentially improving patient outcomes [31,32,33].

The alteration in microbial composition, shifting from initially sterile tracheal aspirates to the predominance of ESKAPEE pathogens, is likely attributed to multiple factors. Prolonged mechanical ventilation, exposure to broad-spectrum antibiotics, and ICU-specific interventions, such as hand hygiene practices, may contribute to the colonization and persistence of multidrug-resistant pathogens. Future research should investigate these temporal shifts by correlating antimicrobial therapies and infection control practices with microbial dynamics. Moreover, exploring the impact of antibiotic stewardship and ICU protocols on shaping microbial dynamics would yield critical insights for preventing the colonization of resistant pathogens.

The ESKAPEE pathogens exhibited increasing resistance over time to critical antibiotics, particularly carbapenems and colistin, which are commonly used as last-resort treatments in ICU settings. Resistance levels were higher overall in tracheal aspirate samples compared to sonication fluids, particularly for *K. pneumoniae* and *A. baumannii*. For these patients, a different antibiotic combination was utilized: Ceftazidime/Avibactam together with Aztreonam. A synergistic effect between the two was noted, effectively countering resistance posed by metallo-β-lactamases and serine β-lactamases, thus restoring susceptibility in multidrug-resistant Klebsiella pneumoniae isolates. These trends suggest that ICU-specific factors, such as antibiotic selection pressures and patient vulnerability, may favor the survival of resistant strains. The increase in carbapenem and colistin resistance specifically raises concerns, as these antibiotics are essential in treating multidrug-resistant (MDR) and extensively drug-resistant (XDR) infections. Given the restricted efficacy of carbapenems and colistin, this finding underscores the need for robust antimicrobial stewardship programs in ICUs. Limiting broad-spectrum antibiotics and focusing on targeted therapy may help to curb the rise of resistant strains.

A significant increase in resistance over time, particularly for carbapenems, necessitates the more precise monitoring of resistance progression to identify at-risk patients and guide early intervention measures. Implementing thorough monitoring at multiple time points in ICU protocols could enhance the optimization of antibiotic therapy and help control the spread of resistance.

The persistent colonization and resistance trends observed, particularly for *A. baumannii*, *K. pneumoniae,* and *S. aureus*, suggest that ICU pathogens may utilize biofilm formation on medical devices as a means of survival. Enhanced ETT management, such as routine cleaning, sterilization, replacement, and antiseptic-impregnated tubes, may help to reduce biofilm-associated infections.

The high persistence rate, as demonstrated by the Markov Chain Model, indicates that once microorganisms are present in the airway, they are likely to persist, despite interventions and treatment. This persistence suggests that the current strategies, while beneficial for some, may not be sufficient for completely eradicating colonization, especially with resistant organisms such as *K. pneumoniae*. If no microorganisms are detected or cleared by T2, there is still a risk of subsequent colonization or contamination, even developing late-onset VAP. This finding underlines the importance of continuous monitoring and potentially repeated interventions in mechanically ventilated patients. Because the effectiveness of interventions is low, we should explore other strategies and interventions to reduce bacterial persistence and improve patient outcomes.

We observed statistically significant differences in the samples after exchanging the endotracheal tube (ETT). Replacing the tube at T2 may help to reduce or change the contamination pattern, potentially preventing biofilm microorganisms from contaminating the tracheal secretions.

The observed link between ETT replacement and changes in microbial composition highlights the crucial role of biofilm management in ICU settings. Regular ETT replacement, paired with improved strategies for disrupting biofilms, could lower the colonization of resistant pathogens and decrease the risk of ventilator-associated pneumonia. These findings emphasize the necessity for customized clinical protocols that combine antimicrobial stewardship with interventions aimed at targeting biofilms.

The increasing resistance to carbapenems, colistin, and vancomycin highlights the need for better antimicrobial stewardship, which includes strict adherence to antibiotic protocols and de-escalation practices whenever possible. Training intensive care unit staff on stewardship principles can help to reduce resistance.

Due to the significant resistance trends among key pathogens, rapid diagnostic tools for real-time resistance profiling can facilitate targeted therapy, reducing the unnecessary use of broad-spectrum antibiotics.

The sample included a significantly higher proportion of male patients (70%), which reflects the demographics of mechanically ventilated ICU patients at the study site. This gender imbalance may affect the generalizability of the findings. However, prior research suggests that gender differences may not significantly influence microbial colonization or resistance patterns in ICU settings [34]. Nevertheless, future studies should aim to use gender-balanced cohorts to confirm these observations and validate the findings.

The variability in ICU-specific antibiotic stewardship protocols represents a critical factor influencing the resistance patterns observed in this study. Antibiotic selection pressures, treatment regimens, and infection control measures can differ significantly across institutions, potentially affecting microbial resistance dynamics and the generalizability of findings. While the present study adhered strictly to the local protocols established at our hospital, these practices may not reflect broader ICU settings. This variability highlights the need for caution when extrapolating these results to other clinical contexts and reinforces the importance of developing standardized protocols that enable cross-institutional comparisons. Additionally, this study’s absence of genotypic profiling limits our ability to identify the molecular mechanisms underlying antimicrobial resistance. Genotypic analyses, such as whole-genome sequencing or the PCR-based detection of resistance genes, could provide valuable insights into the genetic determinants of resistance trends observed, such as the role of extended-spectrum beta-lactamases (ESBLs), carbapenemases, or other resistance mechanisms. Similarly, understanding the genetic factors involved in biofilm formation, such as quorum-sensing genes or biofilm matrix-associated operons, could further elucidate the persistence of pathogens in biofilm-associated infections. Future research should prioritize integrating genotypic profiling and standardized antibiotic stewardship frameworks to enhance the understanding of antimicrobial resistance trends. Such approaches would improve the comparability of findings across diverse ICUs and facilitate the development of targeted interventions to mitigate resistance and biofilm-associated infections. This integration would represent a significant advancement in addressing the global challenge of multidrug-resistant pathogens in ICU settings.

This study has several limitations. It was conducted in a single ICU with a limited sample size, which may affect the generalizability of findings. This study examined a cohort of 30 patients. Although this sample size offers preliminary insights, it limits the statistical power and the generalizability of the findings. To support the observed trends and enhance our understanding of microbial dynamics and resistance patterns in intensive care unit (ICU) settings, it is imperative to conduct larger multicenter studies. Future research should focus on replicating the methodology of this study on an expanded scale to more accurately represent the diverse environments and patient populations present in ICUs. A sampling at only three time points (T1, T2, and T3) might miss transient resistance changes. Still, another diagnostic method for biofilm detection, not sonication, should be used to monitor biofilms more frequently. The lack of genotypic data limits insights into the exact resistance mechanisms. The study did not account for ICU-specific antibiotic stewardship or infection control practices, which could influence resistance patterns, as every other measure taken regarding treatment and infection control followed local protocols and procedures. It might be challenging to distinguish colonization from actual infection as clinical, paraclinical, or imagistic data were not provided to prevent overcharging the paper.

Variability in ICU-specific antibiotic stewardship protocols may influence the observed resistance patterns, as treatment strategies differ across institutions. While this study adhered to local protocols, the findings may not be universally applicable. Furthermore, the absence of genotypic profiling limits insights into the molecular mechanisms underlying resistance trends, such as the presence of specific resistance genes or biofilm-associated gene expression. Future research should incorporate standardized ICU protocols and genotypic analyses to enhance comparability and elucidate the genetic basis of resistance trends observed in ICU pathogens.

## 4. Materials and Methods

This observational longitudinal study focuses on microbial epidemiology in ICU patients undergoing MV admitted to Sibiu County Emergency Hospital in Romania. It is a level I ICU organized on two floors, with 12 single-bed rooms on each floor. In this unit, we treat various medical and surgical pathologies.

The study was created following the protocol published in Medicina, 2023 by Codru et al., but not using all the clinical data [35]. Patients eligible for inclusion in the study are those admitted to the intensive care unit (ICU), of legal age, and who require MV for more than 48 h. The following criteria led to exclusion from the study: patients under the age of 18, situations where informed consent was not adequately obtained (such as cases where the patient or their legal representative has not given consent, has withdrawn consent, or where consent cannot be obtained), instances of improper collection of biological samples, and patients in critical condition for whom the replacement of the endotracheal tube would pose a significant risk to their health or survival.

**Figure 9:**
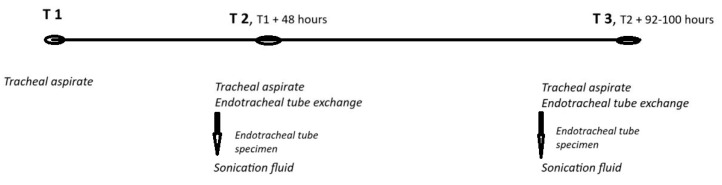
Timeline of microbial monitorization.

Three pivotal time points—T1, T2, and T3—are used to monitor patients during their ICU stay (Figure 9). At T1, which represents either the intubation time or the patient’s admission if already intubated, a tracheal aspirate is collected using a closed-circuit aspiration technique. The second point, T2, occurs 48 h after T1. A second tracheal aspirate is obtained, and the patient’s endotracheal tube (ETT) is replaced. Following the removal of the ETT, a specimen is collected, in an aseptic manner, from the distal 2–3 cm of the tube, which is then placed into a sterile glass container. Thirty milliliters of Ringer’s solution is poured into the container, and the tube is subjected to sonication. This process facilitates the detachment of microbial biofilms or pathogens from the surface of the tube. The sonication fluid is then sent for microbiological analysis, which helps to identify bacterial growth or colonization. T3, the third time point, follows the same procedural steps as T2 but occurs 92–100 h after T2.

Orotracheal intubation cannula specimens are sonicated for 30 min using an ultrasonic bath (BactoSonic14.2, Bandelin GmbH, Berlin, Germany) at a frequency of 42 kHz with a power of 0.22 W/cm^2^. The resulting sonication liquid is then homogenized, and 5–10 mL of the homogenized solution is centrifuged for 5 min at 2500 rpm. The resulting precipitate is inoculated onto culture media and incubated at 37 °C in order to inspect them for bacterial growth.

The bacterial species were identified using traditional biochemical tests (triple sugar iron—TSI, motility indole urea—MIU, Simmons citrate, Gram staining, and methylene blue techniques) and automated identification using the Vitek 2 Compact analyzer (bioMérieux, 376 Chemin de l’Orme, 69280 Marcy l’Étoile, France). In our hospital, antimicrobial susceptibility testing is performed automatically using the Vitek 2 system, which employs specialized cards designed for automated processes. The readings are obtained through a turbidimetric method. The broth microdilution method is reserved for cases involving multidrug-resistant (MDR), extensively drug-resistant (XDR), and pan-drug-resistant (PDR) bacteria.

Antibiotic susceptibility testing was conducted utilizing standardized disk diffusion and minimum inhibitory concentration (MIC) methods following the Clinical and Laboratory Standards Institute (CLSI) guidelines. Resistance patterns for critical antibiotics, such as carbapenems and colistin, were analyzed using automated systems, with manual MIC confirmation performed as necessary. The evaluation of resistance encompassed β-lactams, aminoglycosides, fluoroquinolones, and polymyxins. Furthermore, colistin resistance was validated through broth microdilution techniques.

Sonication was selected as the primary method for biofilm analysis due to its proven efficacy in dislodging bacteria from biofilm structures, ease of implementation, and cost-effectiveness in resource-limited settings. Unlike visual techniques such as confocal microscopy, sonication allows for quantitative microbiological assessment, crucial for evaluating microbial load and resistance profiles. However, alternative methods, such as confocal laser scanning microscopy, could provide additional insights into biofilms’ spatial organization and thickness. Similarly, genotypic approaches, including PCR-based detection, could complement sonication by identifying specific resistance genes and biofilm-related factors. Future studies should consider integrating these methods to provide a more comprehensive understanding of biofilm characteristics and resistance mechanisms.

Sonication was chosen for its proven ability to dislodge bacteria from biofilms in a reproducible and non-destructive manner. Compared to enzymatic treatments or mechanical scraping, sonication offers the advantage of uniform biofilm disruption. However, enzymatic methods may provide complementary insights into biofilm composition, and their use in future studies could enrich our understanding of biofilm-associated resistance [36].

Due to resource constraints, our approach prioritized phenotypic resistance testing, but molecular analyses will be included in future investigations. Incorporating molecular methods, such as PCR and sequencing, to detect resistance genes and mechanisms could provide deeper insights into the persistence of multidrug-resistant pathogens.

The study did not influence the treatment plan and management of the patients discussed in this report. Empirical antibiotic therapy was administered in accordance with our hospital procedures, followed by targeted antibiotics based on microbiological results. All measures to prevent nosocomial infections were implemented for every patient admitted to the ICU. Therefore, any variations in the microbiota can be attributed to changes in the endotracheal tube.

## 5. Conclusions

This study reveals significant antimicrobial resistance trends among ICU pathogens in tracheal aspirates and sonication fluid samples. Detecting resistant bacteria in sonication fluid underscores the role of biofilm formation on endotracheal tubes, which contributes to persistent colonization even after tube exchanges. This biofilm-associated resistance complicates the management of ventilator-associated pneumonia. The observed rise in resistance over time suggests that ICU conditions, such as prolonged ventilation, facilitate the development of hard-to-treat infections. Enhanced protocols for ETT management, including routine sonication and biofilm-disruption techniques, may help to identify and target resistant pathogens, ultimately improving outcomes for ICU patients with VAP.

The rising resistance to critical antibiotics, including carbapenems, colistin, and vancomycin, highlights the urgent necessity for targeted antimicrobial stewardship initiatives in intensive care unit (ICU) environments. The findings emphasize the importance of biofilm-disruption strategies, such as the routine replacement of ETTs and the application of advanced biofilm detection techniques, in reducing the risk of VAP. By addressing the underlying factors that contribute to microbial persistence, these interventions have the potential to improve patient outcomes significantly. Additionally, implementing rapid diagnostic tools for real-time resistance profiling may optimize antibiotic therapy, minimizing the inappropriate use of broad-spectrum antibiotics.

## Data Availability

Data are contained within this article.
